# Cardiac A-lines in fast scan as a sign of pneumopericardium

**DOI:** 10.1186/s13089-019-0123-x

**Published:** 2019-04-25

**Authors:** Julina Md Noor, Elisa A. Eddie

**Affiliations:** 1 Faculty of Medicine, Universiti Teknologi MARA, Sg Buloh Campus, Jalan Hospital, 47000 Sg Buloh, Selangor Malaysia; 2 Emergency & Trauma Department, Hospital Sg Buloh, Sg Buloh, Selangor Malaysia

**Keywords:** Pneumopericardium, Ultrasound, Trauma, Cardiac

## Abstract

**Background:**

Traumatic pneumopericardium is rare and usually results from blunt injury. Diagnosis through clinical and chest X-ray is often difficult. Ultrasound findings of A-line artifacts in the cardiac window may suggest pneumopericardium.

**Case presentation:**

A young man involved in a car accident and sustained blunt thoracic injuries, among others. As part of primary survey, FAST scan was performed. Subxiphoid view to look for evidence of pericardial effusion showed part of the cardiac image obscured by A-lines. Other cardiac windows showed only A-lines, as well. A suspicion of pneumopericardium was raised and CT scan confirmed the diagnosis.

**Conclusions:**

Although FAST scan was originally used to look for presence of free fluid, with the knowledge of lung ultrasound for pneumothorax, our findings suggest that FAST scan can also be used to detect pneumopericardium.

## Background

Pneumopericardium is defined as the presence of air inside the pericardium region and it is commonly found in infants with positive pressure ventilation. In adults, pneumopericardium is a rare and usually results from penetrating or blunt chest injuries or after thoracotomy [1, 2]. Clinical diagnosis is very difficult, if not impossible. Diagnosis is by chest X-ray and is again difficult, the gold standard being computed tomography (CT) scan. Focused assessment of sonography in trauma (FAST) scan is traditionally used to detect pericardial effusion. We report a case of utilizing FAST scan to diagnose pneumopericardium in a trauma patient.

## Case presentation

**Fig. 1:**
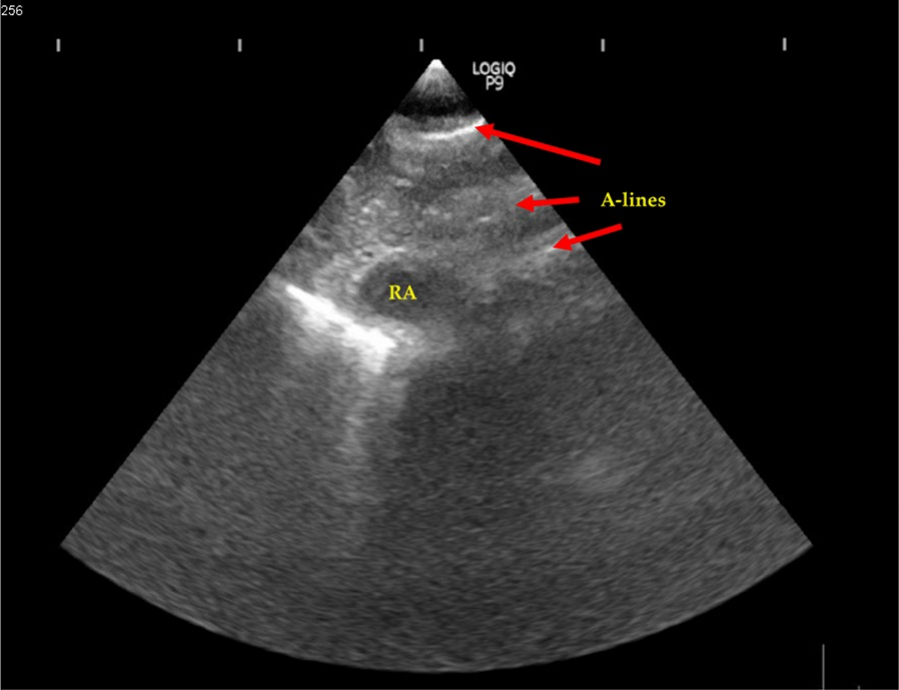
Subxiphoid view showing visible A-lines indicating the presence of air (red arrow)

A 27-year-old male involved in a motor vehicle accident was brought to Emergency Department room with respiratory distress. He was intubated upon arrival due to low Glasgow Coma Scale (GCS) with extensive maxillofacial injuries. Thoracic examination showed reduced air entry at right chest wall region with palpable crepitus on his right neck region due to subcutaneous emphysema from the neck to the anterior chest wall. The heart sound was barely audible. A curvilinear transducer on the right second intercostal ribs shows the absence of sliding signs, suggestive of right pneumothorax. A FAST scan was performed, but, on subxiphoid view of the heart, only the right ventricle is seen during diastole. Part of the cardiac image was obscured by A-lines (Fig. 1). This raised a suspicion of pneumopericardium given the subxiphoid window was showing partly A-lines and the other half of anatomy partially obscured. We proceeded with focused cardiac ultrasound, and only A-lines were visible on parasternal long axis (PLAX), parasternal short axis (PSAX), and apical four chamber (A4C) views.

**Fig. 2:**
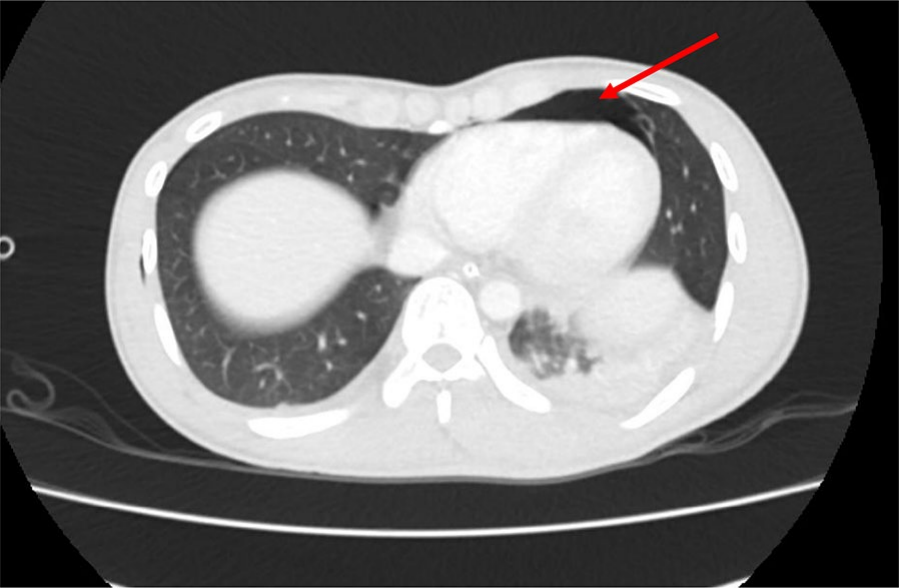
Pneumopericardium on the anterior part on the right ventricle (red arrow)

The patient underwent head and chest CT scan that confirmed the diagnosis of Le Fort II facial bone injury, right pneumothorax, and right pulmonary contusion with pneumopericardium (Fig. 2). The pneumopericardium was treated conservatively, but other injuries were treated accordingly.

## Discussion

The first sonographic evaluation of pneumomediastinum was first described as “air gap sign” [1]. It was described as a broad band of echoes due to accumulation of air obscuring the anterior wall of the heart, with drop out echoes posteriorly. Pneumomediastinum appeared cyclically with the cardiac cycle [3]. In 1994, Allgood et al. [4] described sonographic pneumopericardium findings as the inability to view the heart at the subxiphoid area. This was due to a potential space behind the pericardium that extended inferiorly to the posterior of the pericardium. However, in pneumomediastinum, the heart can be visualized at the subxiphoid area as it was well intact with the diaphragm without obstruction of the air. If there is air in the abdomen (pneumoperitoneum) or if the presence of large gastric bubble, this can obscure the subxiphoid view, thus differential of pneumopericardium and pneumomediastinum can be difficult. The difference in ultrasound findings between pneumopericardium and pneumomediastinum was recently described by Zachariah et al. [5]. In pneumomediastinum, the subxiphoid window remained clear and anatomy is not obscured, suggesting that diaphragm, pericardium, and myocardium were still intact and not obstructed by air. In pneumopericardium, views for parasternal long, parasternal short apical four chambers, and subxiphoid view were poor quality with diffuse A-lines, suggesting air artifact.

Point-of-care ultrasound performed at bedside can lead to a diagnosis that clinical examination alone would not have revealed. Pneumopericardium is not an easy diagnosis to make either clinically or sonographically. Although there is the literature describing pneumopericardium findings, to the best of our knowledge, there is no previous report for trauma cases. FAST scan is a standard ultrasound protocol and is used as an adjunct in primary survey according to the ATLS guidelines. However, the purpose of FAST was to look for free fluid in pericardial and peritoneal regions and, of recent years, presence of haemo- and pneumothorax. With the advancement of knowledge in ultrasound, FAST protocol can be taken to another level. The placement of the probe at the same place as in FAST scan, other pathologies can also be identified, such as atelectasis and diaphragmatic hernia [6, 7]. In our case, partial visualization of the cardiac image with the presence of A-lines on subxiphoid view, coupled with A-lines on all other cardiac views, is highly suggestive of pneumopericardium.

## Conclusion

The role of FAST scan has evolved over the years. As we have now incorporated the detection of pneumothorax and call it extended focused assessment of sonography in trauma (E-FAST), treating physicians must be aware of other possible diagnosis that can be detected with FAST scan. We suggest that the presence of cardiac A-lines in subxiphoid cardiac view should alert the physician to the probability of pneumopericardium.

## Abbreviations

FAST: focused assessment of sonography in trauma
CT: computed tomography
GCS: Glasgow coma scale
PLAX: parasternal long axis
PSAX: parasternal short axis
A4C: apical four chamber
ATLS: advanced trauma life support
E-FAST: extended focused assessment of sonography in trauma
